# Scalable ultrafast epitaxy of large-grain and single-crystal II-VI semiconductors

**DOI:** 10.1038/s41598-020-59083-3

**Published:** 2020-02-12

**Authors:** Eric Colegrove, David S. Albin, Helio R. Moutinho, Mahisha Amarasinghe, James M. Burst, Wyatt K. Metzger

**Affiliations:** 1 National Renewable Energy Laboratory, Golden, CO 80401 USA; 2 Department of Physics, University of Illinois at Chicago, Chicago, IL 60607 USA

**Keywords:** Semiconductors, Materials for devices, Materials science, Solar cells

## Abstract

A general problem for semiconductor applications is that very slow deposition on expensive single-crystal substrates yields high crystalline quality with excellent electro-optical properties, but at prohibitive costs and throughput for many applications. In contrast, rapid deposition on inexpensive substrates or nanocrystalline films yields low costs, but comparatively inferior crystallinity, carrier transport, and recombination. Here, we present methods to deposit single-crystal material at rates 2–3 orders of magnitude faster than state-of-the-art epitaxy with low-cost methods without compromising crystalline or electro-optical quality. For example, single-crystal CdTe and CdZnTe films that would take several days to grow by molecular-beam epitaxy are deposited in 8 minutes by close-spaced sublimation, yet retain the same crystalline quality measured by X-ray diffraction rocking curves. The fast deposition is coupled with effective n- and p-type *in-situ* doping by In, P, and As. The epitaxy can be extended to nanocrystalline substrates. For example, we recrystallize thin CdTe films on glass to deposit large grains with low defect density. The results provide new research paths for photovoltaics, detectors, infrared imaging, flexible electronics, and other applications.

## Introduction

For decades, semiconductor applications have been both enabled and limited by available deposition methods and their corresponding electro-optical quality and cost tradeoffs^1^. Single-crystal semiconductors generally have excellent electro-optical properties but are deposited relatively slowly, limited to small areas, and require expensive single-crystal substrates. In contrast, amorphous, nanocrystalline, and polycrystalline materials can be produced more quickly with orders-of-magnitude lower cost over large areas on inexpensive substrates such as glass, metal foils, and polymers, but generally have inferior electro-optical properties, carrier recombination, and transport^1–3^.

**Figure 1:**
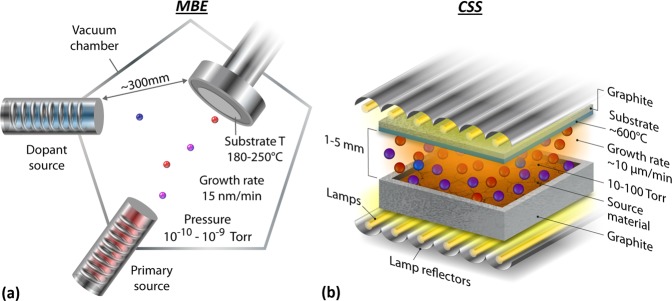
Schematics of MBE **(a)** and CSS **(b)** indicating the differences in design, pressure, temperature, and deposition rates.

A number of fundamental physical mechanisms give rise to the slow rates and high costs associated with single-crystal deposition, including the need for: (1) sufficient time for atoms to bond in appropriate lattice sites within the limits of chemi-adsorption rates, surface transport, and flux rate; (2) single-crystal substrates and careful surface preparation to provide a crystalline structure and pristine surface for nucleation; (3) high-purity source materials to eliminate unintended impurities that cause improper bonding and structural defects; and (4) ultra-high vacuum (UHV) and/or ultra-high purity (UHP) gases to prevent impurities that cause poor nucleation and/or deteriorate electro-optical properties. Figure 1(a) shows a schematic representation of a molecular-beam epitaxy (MBE) system using the low-temperature, low-flux, and UHV conditions typical of most single-crystal epitaxial processes.

New methods to overcome these traditional obstacles^2–4^ can lead to seminal shifts in throughput and costs for a number of technologies such as infrared imaging, flexible electronics, photovoltaics (PV), and detectors^5–7^. For example, in PV, polycrystalline CdTe is deposited at rates on the order of microns per second with low-vacuum equipment to enable high throughput to produce millions of panels annually^8–10^. One fast thin-film deposition method is close-spaced sublimation (CSS), with the process shown schematically in Fig. 1(b). The resulting grain size after CSS deposition is typically several hundred nanometers initially, which leads to significant interface and grain boundary recombination and limits CdTe PV efficiency^11,12^. New approaches to overcome such limitations can enable PV to further undercut the electricity costs of conventional fuels.

Conversely, single-crystal III-V epitaxial multijunction solar cells have reached record cell efficiencies of 47%^9,13,14^, but the high-vacuum equipment and single-crystal substrates are very expensive and reduce market penetration. Furthermore, substrates are just several inches across, and the deposition rates are on the order of μm/h, leading to slow throughput. Consequently, this technology has remained limited to applications where cost is less critical such as space PV^15^. Current efforts are examining new processes to expand the breadth of applications while retaining the outstanding performance associated with MBE and metalorganic vapor-phase epitaxy (MOVPE) III-V multijunctions^16,17^. These applications include overcoming single-junction efficiency limits at costs and throughput that are practical for broader PV, as well as infrared and high-energy detector technologies^6,7,16–21^.

In this work, we demonstrate that high quality epitaxy can be achieved with low-cost equipment scalable to large areas to enable single-crystal CdTe and CdZnTe deposition at rates of ~10 μm/min, which is 2 to 3 orders of magnitude faster than state-of-the-art epitaxial methods. Beyond single crystals, fast epitaxy can be extended to polycrystalline films grown on non-crystalline substrates (e.g. glass, polymers, and metal foil). As an example, nanocrystalline CdTe films are first recrystallized on glass, this then forms the template to seed large-grain CSS epitaxial growth with low defect density.

## Single Crystal Epitaxy

Epitaxial crystal deposition can be divided into two sub-categories: homoepitaxy and heteroepitaxy^3^. In homoepitaxy, a single crystal with the same composition provides an ideal seed layer. Heteroepitaxy is relatively straightforward for lattice-matched compositions, but it is much more difficult for lattice-mismatched layers, where the accumulated strain can lead to highly defective regions.

In MBE, high quality epitaxial deposition is facilitated by low flux at the crystal surface, thereby allowing more time for flux species to dissociate, migrate, and bond in appropriate lattice sites^19^. Figure 1(a) provides a rough MBE schematic. In this approach, state-of-the-art vacuum equipment is required to maintain the background ambient pressure to 10^−9^–10^−10^ torr; this provides outstanding impurity control to avoid both nucleation defects and carrier compensation. It also enables the flux of atoms from the primary source, typically a CdTe effusion cell, to be extremely low. Too much flux can cause the Cd and Te atoms at the surface to have insufficient time to bond in the appropriate locations, thus resulting in higher defect densities and possibly polycrystalline material. If the substrate temperature is raised too high, then atoms at the crystal surface sublime at a deleterious rate, leading to film loss in lieu of crystal growth. Dopant atoms are introduced from a separate source, and dopant flux rates are varied to incorporate target concentration levels. Because of these physical constraints, CdTe MBE is generally performed at a substrate temperature of 180°–250 °C. The pressure produced by the flux of Cd and Te molecules, represented by red and purple spheres in Fig. 1(a), known as the beam equivalent pressure, is about 10^−6^ torr, resulting in deposition rates of about 15 nm/min^22^.

Figure 1(b) shows the stark contrast of CSS deposition. In this approach, a CdTe source is typically about 1.5 in × 1.5 in across in our laboratory, but it is scalable to meters squared in manufacturing. The source is typically heated to temperatures ranging from 600°–700 °C. The substrate temperature may be varied from 200°–600 °C^23,24^. Background pressures, typically about 10–100 torr, can control the vapor diffusion rate to the surface, and hence, the flux rate. The low-vacuum conditions enable extremely inexpensive designs, such as the use of lamps to heat graphite boats in a quartz container backfilled with ambient gases (orange glow in Fig. 1(b)). To enable depositing enormous amounts of material at the rates required for terrestrial PV, deposition rates are on the order of μm/min. Traditionally, films are deposited on glass and nanocrystalline substrates, and the resulting films have grains on the order of several hundred nanometers at the initial interface that coalescence into larger grains of 1–2 microns. As-deposited films have lifetimes on the order of tens or hundreds of picoseconds and hole density less than 10^14^ cm^−3^, which severely limit performance^12^. Post-deposition methods are then applied to try to improve these initial electro-optical properties as much as possible; but they introduce compensated defect chemistries, delamination, and other difficult material challenges. The premise is flawed for the high performance required for modern photovoltaics—but the throughput and costs are ideal.

Here, experiments are examined to combine the throughput and low cost of CSS with the high crystalline quality of traditional MBE. To examine both the role of the substrate in CSS-deposited films, as well as potential applications for Si/CdTe tandem PV, a heteroepitaxial CdTe substrate layer was deposited by MBE on a Si(211) wafer using a thin pseudomorphic ZnTe(211) interface layer to mitigate strain between the lattice-mismatched materials. The slightly different Si and CdTe colors indicate about a 4° tilt between the Si(211) orientation and the CdTe(211) orientation^22^. After a CdTe seed layer had been established, the deposition was stopped, and the sample was transferred to the CSS system.

**Figure 2:**
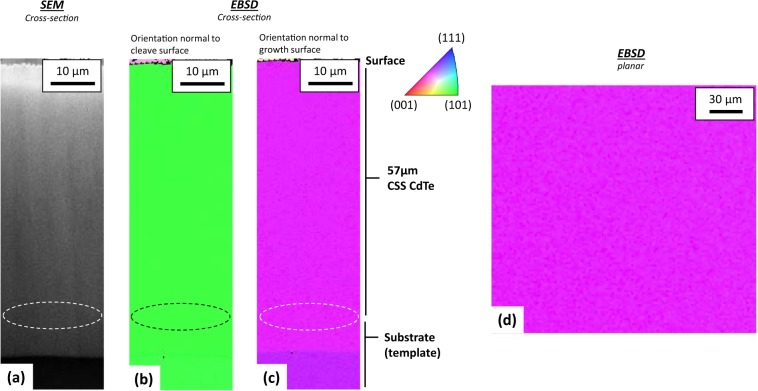
Cross sectional SEM **(a)** and EBSD **(b,c)** of films grown in part by CSS CdTe at high rates oriented normal to the cleave and growth surfaces, respectively. Planar EBSD of the top surface **(d)**. Uniform color of the EBSD indicates a single crystal orientation.

A 57-μm thick homoepitaxial single-crystal CdTe film was then grown in about 8 minutes on this layer. Figure 2 shows scanning electron microscopy (SEM) and electron backscatter diffraction (EBSD) data for a cross-section of the CdTe film. Depositing films of this thickness using conventional MBE takes about 2.5 days—rather than 8 minutes. A dashed line oval illustrates the general regrowth region. EBSD provides 3D crystallographic orientation data enabling examination of the interface from multiple perspectives. For the two orthogonal perspectives shown, there are no observable interface features evidenced by EBSD or corresponding SEM between the seed layer and the CSS film. Figure 2(d) illustrates the planar view of the top portion of the CdTe film after ion milling. As-deposited films can have surface blemishes, but either ion milling or a brief bromine methanol removes these blemishes, indicating they do not run throughout the film thickness and are likely caused by contamination after growth. Relative to polycrystalline films, the image indicates the large area over which single crystallinity is achieved.

Several factors enable the fast epitaxy. The CSS substrate temperature implemented in these studies is ~600 °C. This is achievable because the CSS background pressure (10–100 torr) and high flux create an overpressure that confines Cd and Te re-evaporated atoms near the growth surface for significantly longer periods of time, whereas the extremely high vacuum and low flux in MBE do not. The additional thermal energy at the crystal surface greatly facilitates Cd and Te nucleation at appropriate lattice sites where the bond strength is high—24 kcal/mol^25^. When dislocations and stacking faults resulting from improper bonding do occur, the high substrate temperature can provide sufficient thermal energy to restore proper lattice configurations, migrating and annihilating dislocations as the crystal is nucleating and growing. In conventional MBE, short high-temperature cycles with Te overpressure are used to produce the same type of dislocation migration and annihilation, just much more slowly^22^. CdTe is also a binary alloy that sublimates congruently^26^, enabling the correct and constant source-flux stoichiometry for nucleation and subsequent crystal growth.

**Figure 3:**
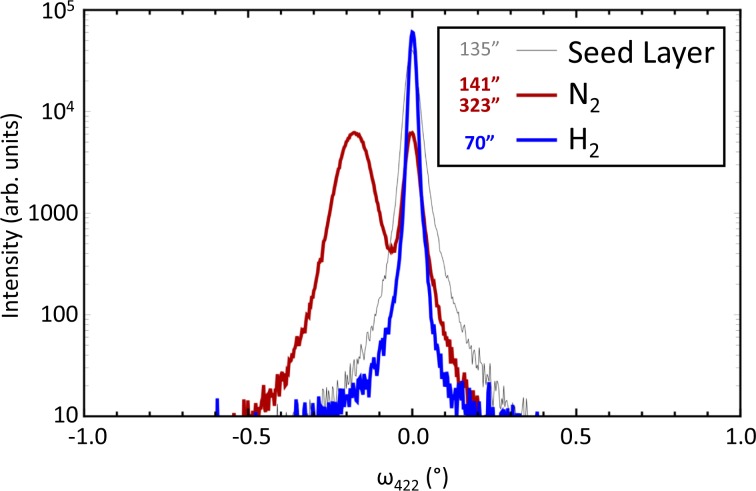
HR-XRD rocking-curve scans for CSS epitaxial films. The seed layer is shown in gray, and the CdTe films deposited in nitrogen (1–2 μm) and hydrogen (22 μm) are shown in red and blue, respectively. The FWHM for each film is shown in the legend.

Another critical factor for the epitaxy is the growth ambient. Most traditional CSS deposition uses inert gases such as He or N_2_ coupled with some oxygen. Figure 3 shows high-resolution X-ray diffraction (HR-XRD) rocking-curve scans of the (422) peak for (211)-oriented epitaxial CdTe films deposited by CSS on single-crystal CdTe templates. Crystalline quality is often gauged by the full width at half maximum (FWHM) of XRD curves. When an inert ambient such as nitrogen or helium that does not properly remove surface oxides was introduced, we found that only a thin nanocrystalline or amorphous film (0.1–2 μm thick) was deposited (Fig. 3, red curve), indicating poor epitaxy. Naturally, adding oxygen will exacerbate oxide formation. However, an ambient containing hydrogen strips surface oxide formation prior to and during deposition. Here, the hydrogen containing ambient accelerates deposition rates by a factor of ten, greatly enhances material quality, and is critical for fast epitaxy^27^. For example, the blue curve in Fig. 3 illustrates a high-quality CSS epitaxial film with a FWHM of 70′′ formed by growing in a hydrogen ambient.

## Fast Single Crystal Epitaxy Coupled With Doping

Rapidly deposited undoped single-crystal CdTe films can be useful for a variety of applications, such as X-ray and γ-ray detectors^28^. Other applications—such as high-performance PV, light-emitting diodes, and lasers—require effective doping^5,11,29,30^. In MBE applications, some dopants are introduced by valve cracker cells (e.g., dopant source in Fig. 1(a)) operating near 900 °C to disassociate dopant tetramers into dimers (e.g., As_4_ → 2As_2_), with the latter population increasing with the cracker temperature. This is critical to allow effective dopant adsorption, disassociation, and incorporation within the growing crystal lattice, and it is not clear that fast deposition methods can maintain effective dopant flux, adsorption, and incorporation kinetics^31^.

Here, we examined whether single crystals grown at high rates by CSS could achieve effective n-type and p-type doping by simply embedding In, As, and P in the source material^28^. CdTe source material contained ~1 × 10^18^ cm^−3^ As or P concentrations according to ICP measurements. After deposition, the films contained ~1 × 10^17^ cm^−3^ As or P concentrations based on dynamic secondary ion mass spectroscopy measurements. These samples were subjected to rapid thermal processing (RTP) at 575 °C for 1 min to place the GrV atoms on Te sites. Hall measurements indicated hole densities from 3 × 10^15^ to 5 × 10^15^ cm^−3^. Films with similar incorporation levels were also deposited from CdTe source material containing 5 × 10^18^ In atoms cm^−3^. Here, as-deposited films with no further activation achieved free-electron densities of 5 × 10^15^ cm^−3^. Exhaustive doping work was not executed and future work can likely improve upon these results. The experiments indicate that fast epitaxy by close-spaced sublimation can be coupled with incorporating and activating dopants. The next section indicates doping could also be achieved with heteroepitaxy of CdZnTe:P on Si/CdTe templates.

## Fast Single Crystal Heteroepitaxy and Doping

**Figure 4:**
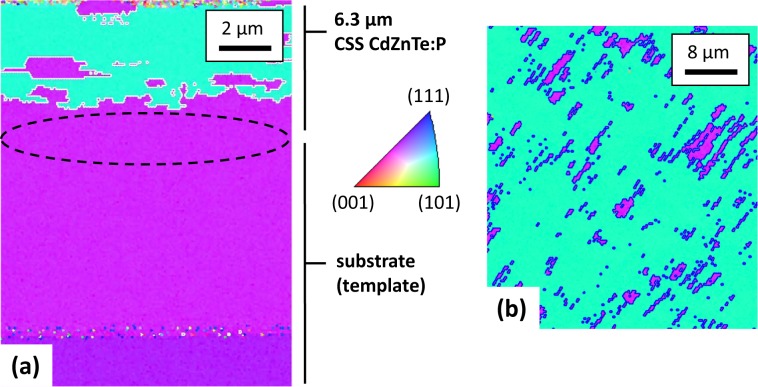
EBSD data of CSS CdZnTe:P on a MBE CdTe template. Cross-section **(a)** and planar **(b)** orientation maps showing the <011> and <112> twinned surface.

CdZnTe is a common detector material and an important potential top-cell material for Si-based tandem PV because it has a higher bandgap than CdTe^32^. In common epitaxy, stoichiometry shifts causing just 0.5% lattice mismatch typically result in extended dislocations in the deposited film. Nonetheless, here, we attempted to grow CdZnTe on CdTe at fast rates on the order of 10 μm/min. Figure 4 shows EBSD cross-sectional and planar images for a phosphorus-doped Cd_0.94_Zn_0.06_Te layer grown by CSS on a MBE CdTe template. The lattice mismatch^33^, together with potentially imperfect flux stoichiometry, results in substantial twinning of the epitaxial film. Yet, this film still has just two primary orientations with coincidence site lattice Σ3 boundaries and no randomly oriented grain boundaries (GBs)^34^. The CdZnTe source material contained 9 × 10^17^ cm^−3^ P atoms. The deposited films achieved 1 × 10^16^ to 5 × 10^16^ cm^−3^ hole density as measured by Hall after RTP for 1 minute at temperatures of 550 °C–575 °C^31,35,36^.

## Fast Polycrystalline Epitaxy

Thin films are often deposited on non-crystalline substrates for applications such as flexible electronics and PV. In thin-film solar cells, the absorber is typically deposited on a nanocrystalline substrate formed by a buffer, transparent conducting oxide, and glass film stack. Lightweight applications may seek to replace the glass with a polymer or metal foil^5,37–41^. If high-quality columnar grains can be deposited with diameters significantly larger than the film thickness, then the grain size can enable electron transport and recombination that is largely independent of GBs^42^. For example, Kanevce *et al*.^11^ computationally simulated the effect of grain size on the open-circuit voltage (V_OC_) and resulting cell efficiency for typical thin-film CdTe PV devices as a function of GB recombination velocity (S_GB_). Assuming a typical value of S_GB_~10^5^ cm/s^43–48^ and bulk lifetime of tens of ns, then an increase in grain size from 1 to 10 microns can improve V_OC_ from about mid-800 mV to nearly 1000 mV and efficiency from 15% to 25%^11^.

Furthermore, for CdTe applications, CdCl_2_ treatments are generally required to passivate GBs, however the Cl can cause carrier compensation^49^. For example, for years polycrystalline films fabricated with CdCl_2_ treatments and Cu by diverse methods at different institutions across the world were limited to hole density on the order of 10^14^ cm^−341^. Recent polycrystalline GrV solar cells show 100 times the hole density and significantly better dopant stability than Cu^30^. At the same time, they currently have activation on the order of 0.3–3% whereas single crystals doped with GrV elements can achieve 50% activation, even though dopants do not appear to accumulate at the grain boundaries^30,50,51^. The low activation levels in polycrystalline solar cells can introduce potential fluctuations that limit performance^30^. If a thin template layer can be established by a CdCl_2_ treatment or another process, the remainder of the film perhaps could be grown by low cost epitaxy without Cl, thereby opening up paths for distinct doping approaches with less compensation while retaining larger grains.

**Figure 5:**
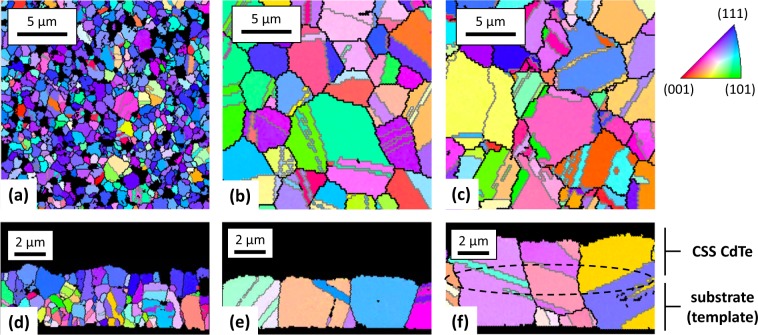
EBSD data showing CdTe grain growth and resulting from high-temperature CdCl_2_ treatment and subsequent CSS epitaxy. Planar **(a)** and cross-sectional **(d)** EBSD for the initially deposited film; planar **(b)** and cross-sectional **(e)** EBSD for the recrystallized film; and planar **(c)** and cross-sectional **(f)** EBSD for the regrown CdTe film on the recrystallized template.

Historically, CdTe grain size has been moderately controlled by growth conditions such as higher substrate temperature, total pressure, oxygen partial pressure, and/or post-deposition CdCl_2_ or MgCl_2_ annealing^39,40,52–56^. Fig. 5(a,d) show planar and cross-sectional EBSD data for a typical CSS film—the grains are small, particularly near the interface, with an average size of several hundred nm. GBs are typically defective and will naturally induce electrostatic potentials distinct from the bulk; horizontal GBs (e.g. parallel to the plane of the metallurgical junction) create energetic barriers to carrier transport across the film^55^. GBs of every orientation that are not Σ3 (black lines in Fig. 5(a–f)) will cause recombination^52^. Consequently, a preponderance of small, randomly oriented grains near the interface can hinder device performance^11,57^.

Here, we examine a proof of concept that epitaxy can be performed on large grain templates. Future work will examine different methods and approaches to achieve similar templates with thinner layers and different approaches. The large-grain templates were established by aggressively establishing the CdTe–CdCl_2_ eutectic to recrystallize small CdTe grains nucleated on CdS nanocrystalline films on glass/TCO substrates^54^. The liquid phases resulting from the eutectic where CdCl_2_ concentrations are high, at and around GBs, accelerate the Ostwald ripening grain-growth mechanism^51^. To realize this, films are heated to 500 °C in close proximity to a glass cover plate to help prevent outgassing and delamination. Figure 5(b,e) indicate that the recrystallization forms columnar grains ranging up to 10 μm. The grains do not have high intragrain defect density.

The recrystallized large-grain film is used as a test template to seed large-grain CSS deposition using the rapid epitaxial methods described earlier. Figure 5(c,f) demonstrate remarkably that even the twins and GBs grow seamlessly on the template without any apparent interface^4,58^. Sister samples were measured by time-correlated single-photon counting and indicate approximately a tenfold increase in lifetime relative to the as-deposited samples.

Fast, scalable, low-cost epitaxial growth sets the stage to examine a number of nucleation, melt, and other processes to seed large grains, and it can be extended to other compositions and materials. For example, CdMgTe and CdZnTe can achieve the ideal bandgap (1.7–1.8 eV) energy for top cells in Si/II-VI tandem PV^32,59^. However, the Mg and Zn migrate out of the semiconductor during CdCl_2_ treatment and reduce the bandgap back to 1.5 eV^59^. By first forming large-grain seed layers with the approach above, high-quality CdMgTe or CdZnTe grains with long lifetimes could be deposited without Cl, thus circumventing CdMgTe and CdZnTe decomposition. This could then be extended to Si/II-VI tandem applications.

## Conclusions

Single crystal epitaxy with scalable low cost equipment at rates 2–3 orders of magnitude faster than state-of-the-art MBE is enabled by high temperatures, high flux rate, and critically a reducing ambient. For example, CdTe single crystals that would take a few days to grow by MBE are deposited in just 8 minutes by close-spaced sublimation. Yet, they retain high crystalline quality as indicated by X-ray rocking curves, and they can be coupled with effective n- and p-type doping. The results can be extended to heteroepitaxy and polycrystalline structures, providing new research paths for photovoltaics, detectors, infrared imaging, flexible electronics, and other applications.

## Methods

### Fabrication

Heteroepitaxial depositions of CdTe on 3′′-diameter Si(211) wafers were performed in a Veeco GEN930 MBE system. RCA oxides are desorbed at greater than 1000 °C, exposing a clean Si surface that is passivated with a monolayer of arsenic as the substrate cools. This is followed by migration-enhanced epitaxy of a 15-nm pseudomorphic ZnTe layer and finally by nucleation of the CdTe layer between 180 °C and 250 °C, with deposition proceeding at a rate of about 1 μm/h. *In-situ* anneals are performed after about every 2 μm of growth to improve material quality^22^. Background pressure for the MBE system is on the order of 10^−10^ torr. Source materials for these films were 7N purity and supplied by 5N Plus. These films were deposited to thicknesses ranging from 3 to 10 μm and were used as single-crystal templates for MBE or CSS deposition.

Initial polycrystalline CdTe films were deposited with a custom-built CSS system on superstrate stacks consisting of glass, 500 nm of metal-organic chemical vapor deposition polycrystalline SnO_2_:F, and about 100 nm of sputtered CdS:O^23^. The background pressure for the CSS system is on the order of 10^−2^ torr, and deposition ambient pressures ranged from 10 to 50 torr using gas mixtures of H_2_, He, N_2_, and O_2_. During deposition, the substrate temperature was 600 °C and the growth rate varied from 1–10 μm/min. Epitaxial regrowth experiments used the same processing conditions as those described for the initial film depositions. CdTe containing In and CdZnTe containing P were provided by Washington State University^41^. CdCl_2_ treatments were performed in CSS systems with 4N-purity anhydrous beads to generate a vapor overpressure.

### Characterization

Crystal orientation and quality were assessed by electron backscatter diffraction inverse-pole figure mapping. In this technique, different crystalline orientations—and thus, grains—are resolved by electron diffraction and illustrated by different colors; black regions correspond to regions where the software failed to resolve the crystallinity. For cross-sectional images, the colors correspond to crystal orientation normal to the growth direction, not the plane of the cross-section. EBSD was measured by an FEI Nova 630 NanoSEM with an EDAX Pegasus/Hikari A40 system. To avoid shading and other sample-roughness artifacts, a JEOL cross-section polisher smoothed planar and cleaved cross-section samples. Time resolved photo luminescence (TRPL) measurements were carried out using femtosecond laser pulses fired at 1.1 MHz with sub-bandgap photons of 1.11 eV. CdTe photoluminescence emission was isolated using an 819-nm bandpass filter with 44-nm bandwidth, and high temporal-resolution decay curves were generated using time-correlated single-photon counting. HR-XRD was measured by a Rigaku SmartLab system. Dopant incorporations levels were measured by dynamic secondary ion mass spectrometry profiling at EAG Laboratories. A BioRad HL5500PC system was used for Hall measurements in the van der Pauw configuration.
